# Interleukin 34: a new modulator of human and experimental inflammatory bowel disease

**DOI:** 10.1042/CS20150176

**Published:** 2015-05-15

**Authors:** Stephanie Zwicker, Gisele L. Martinez, Madeleen Bosma, Marco Gerling, Reuben Clark, Mirjam Majster, Jan Söderman, Sven Almer, Elisabeth A. Boström

**Affiliations:** *Department of Dental Medicine, Division of Periodontology, Karolinska Institutet, Alfred Nobels Allé 8, SE-141 52, Huddinge, Sweden; †Department of Cell and Molecular Biology, Karolinska Institutet, P.O. Box 285, SE-171 77, Stockholm, Sweden; ‡Department of Biosciences and Nutrition, Karolinska Institutet, Novum, SE-141 52, Huddinge, Sweden; §Division of Medical Diagnostics, Ryhov County Hospital, Building E3 Level 4, SE-551 85, Jönköping, Sweden; ║Department of Medicine Solna, Karolinska Institutet, SE-171 76, Stockholm, Sweden; ¶GastroCentrum, Karolinska University Hospital Solna, SE-171 76, Stockholm, Sweden

**Keywords:** colon epithelial cells, CSF-1, IBD, IL-34, intestine, macrophage, AD, atopic dermatitis, CD, Crohn’s disease, CSF-1, colony-stimulating factor 1, CSF-1R, CSF-1 receptor, DSS, dextran sodium sulfate, IBD, inflammatory bowel disease, IKKβ, IκB (inhibitor of NF-κB) kinase β, IL, interleukin, LSD, least significant difference, NF-κB, nuclear factor κB, qPCR, quantitative real-time PCR, RA, rheumatoid arthritis, TNF-α, tumour necrosis factor α, UC, ulcerative colitis

## Abstract

IBD (inflammatory bowel disease), where CD (Crohn's disease) and UC (ulcerative colitis) represent the two main forms, are chronic inflammatory conditions of the intestine. Macrophages play a central role in IBD pathogenesis and are regulated by major differentiation factors such as CSF-1 (colony-stimulating factor 1) in homoeostasis and inflammation. IL (interleukin)-34 has recently been discovered as a second ligand for CSF-1R (CSF-1 receptor). However, expression and involvement of IL-34 in IBD remain unknown. In the present paper, we investigated the expression of *IL34*, *CSF1* and their shared receptor *CSF1R* in normal human ileum and colon, in inflamed and non-inflamed tissues of CD and UC patients, and in a mouse model of experimental colitis. We found distinct expression patterns of *IL34* and *CSF1* in ileum and colon, with higher *IL34* in ileum and, in contrast, higher *CSF1* in colon. Furthermore, *IL34* and *CSF1* expression was increased with inflammation in IBD patients and in experimental colitis. In humans, infiltrating cells of the lamina propria and intestinal epithelial cells expressed IL-34, and TNF-α (tumour necrosis factor α) regulated IL-34 expression in intestinal epithelial cells through the NF-κB (nuclear factor κB) pathway. These data demonstrate the expression pattern of IL-34 in ileum and colon and suggest IL-34 as a new modulator of inflammation in IBD.

## Abstract 2

The macrophage differentiation factor interleukin-34, produced by intestinal epithelial cells, is up-regulated in patients with inflammatory bowel disease, and may be a novel modulator of intestinal inflammation.

## INTRODUCTION

IBD (inflammatory bowel disease) is a group of chronic inflammatory conditions of the intestine where CD (Crohn's disease) and UC (ulcerative colitis) represent the two main forms. Genetic predisposition, environmental factors and dysregulated immune responses, as well as impaired intestinal epithelial function, sustain the inflammation; however, the complete pathogenesis remains unknown [1–3].

Macrophages are central to intestinal homoeostasis, being strategically positioned in the subepithelial lamina propria to clear microbes that breach the epithelial barrier. Tissue macrophages originate from the mononuclear phagocyte lineage that differentiate under the control of the transcription factor PU.1 through a series of events that ends with the binding of CSF-1 (colony-stimulating factor 1) to its tyrosine kinase receptor CSF-1R (CSF-1 receptor) (reviewed in [4–7]). In the non-inflamed gut, terminally differentiated macrophages can be distinguished from other tissue macrophages and do not become activated or produce pro-inflammatory cytokines in response to bacteria or bacterial products [8]. However, in the presence of inflammation, newly recruited monocyte-derived macrophages increase in numbers, and display phenotypical and functional differences. Compared with the non-inflamed bowel, differences in Fc and complement receptors that mediate cellular activation and enhanced secretion of TNF-α (tumour necrosis factor α) are evident [9–12].

On the basis of the finding that CSF-1R-deficient mice show a more severe phenotype than mice lacking CSF-1, a second functionally overlapping ligand for the receptor had been proposed [13]. IL (interleukin)-34 was identified as an additional ligand through a functional screen of extracellular proteins [14]. Like CSF-1, IL-34 is a key regulator of the differentiation, proliferation and survival of cells from the mononuclear phagocytes lineage [14,15]. Highly conserved, IL-34 and CSF-1 are structurally related [16], but differences have been reported in their expression patterns in embryonic and adult tissue. IL-34 is expressed in the early development of the embryonic brain before the expression of CSF-1, and more abundantly in postnatal and adult brain in non-overlapping regions to CSF-1 [15,17]. The main sources of IL-34 in adult mice are neurons and skin keratinocytes [18]. However, other cellular sources and the regulation of IL-34 under inflammatory conditions and in humans have been sparsely studied.

The involvement of IL-34 in RA (rheumatoid arthritis) and other inflammatory conditions has caught recent interest. IL-34 is expressed in RA synovium where it relates to the synovitis severity [19] and is elevated in serum and synovial fluid of RA patients [19]. Furthermore, IL-34 expression is up-regulated in inflamed salivary glands from patients with Sjögren's syndrome [20]. Moreover, synovial and gingival fibroblasts produce IL-34 in response to TNF-α and IL-1β through NF-κB (nuclear factor κB) and JNK (c-Jun N-terminal kinase) pathways, and IL-34 can substitute for CSF-1 in osteoclastogenesis [19,21]. Furthermore, blockade of TNF-α in RA patients by infliximab reduces IL-34 expression [22]. Until now, studies on the expression and regulation of IL-34 in the intestine and its potential involvement in IBD pathogenesis are lacking.

The aim of the present study was to assess the expression of IL-34, CSF-1 and their joint receptor, CSF-1R, in human ileum and colon under normal conditions. Furthermore, we investigated their regulation with inflammation in human IBD and in experimental colitis in mice. We also investigated the localization of IL-34 in the intestine and hypothesized that IL-34 expression was regulated by TNF-α through NF-κB, a pivotal cytokine in IBD pathogenesis. We assessed further the expression of IL-34, CSF-1 and pro-inflammatory factors in healthy and IBD monocytes, and investigated the expression of cytokines in IL-34- compared with CSF-1-differentiated macrophages. Together, our findings provide evidence for the expression pattern and a potential role for IL-34 in IBD.

## MATERIALS AND METHODS

### Human IBD cohort

A total of 52 adult patients investigated for a known IBD diagnosis or in the work-up for suspected gastrointestinal disorders (Supplementary Table S1) were subjected to colorectal and ileal mucosal biopsies during routine endoscopy. In addition, 33 patients not afflicted with IBD and without intestinal inflammation or pathological findings were included as non-inflamed non-IBD controls. Biopsies were collected in parallel to, and from the same locations as, biopsies for histopathological assessment. Each biopsy was classified as ‘inflamed’ or ‘non-inflamed’ on the basis of a composite evaluation of macroscopic findings assessed by one experienced endoscopist (S.A.) and routine histopathologic assessment. Biopsy specimens for RNA purification were immersed in RNA*later* RNA stabilization reagent (Qiagen) and stored at 4°C overnight and thereafter at −20°C until RNA purification. The study was carried out in accordance with the Declaration of Helsinki (2008) of the World Medical Association and approved by the Regional Ethical Review Board in Linköping, Sweden (Dnr 2011/201-31). Written informed consent was obtained from all participants.

### Macrophage cultures

PBMCs (peripheral blood mononuclear cells) were isolated from buffy-coated blood using Ficoll-Hypaque gradient centrifugation (BD Diagnostics), followed by monocyte isolation using the EasySep Human monocyte enrichment kit without CD16 depletion (StemCell Technologies), according to the manufacturers’ protocols. Then, 5×10^5^ monocytes/well were plated in six-well plates with complete RPMI 1640 medium supplemented with 50 ng/ml CSF-1 or IL-34 (BioLegend) for 8 days to generate macrophages.

### DSS-induced colitis in mice

Colitis was induced by administration of 3% DSS (dextran sodium sulfate) (molecular mass 40 kDa, #DB001, TdB Consultancy) with the drinking water, provided *ad libitum* for 5 days. Untreated control mice received tap water only. The mice, >10 weeks of age, were housed in groups of three to ten at 20–22°C in a 12 h light/12 h dark cycle and fed with standard chow diet. All animal procedures were in compliance with protocols approved by local government authorities (The Board of Agriculture, Experimental Animal Authority, Stockholm, Sweden). Body weight was measured daily.

### Colon epithelial cells

Caco-2 cells (A.T.C.C., Manassas, VA, U.S.A.) were cultured in DMEM (Dulbecco's modified Eagle's medium) supplemented with 10% (v/v) FBS (Gibco-BRL/Life Technologies), 1% NEEA (Gibco-BRL/Life Technologies) and 1% GlutaMAX™ (Invitrogen) at 37°C and 5% CO_2_. Cells were seeded in 24-well plates and, after attachment for 48 h, the medium was changed and the cells were incubated in the absence (controls) or presence of TNF-α (1–100 ng/ml, BioLegend) or the signalling pathway inhibitors celastrol (catalogue number 3203; Tocris) or IMD 0354 (catalogue number 2611; Tocris) 1 h before stimulation with TNF-α. Cell lysates were subjected to RNA isolation.

### RNA isolation, cDNA synthesis and qPCR (quantitative real-time PCR)

Human intestinal biopsies were homogenized using a TissueRuptor and disposable probes (Qiagen). RNA was purified using the AllPrep DNA/RNA mini kit (Qiagen) according to the manufacturer's instructions, either manually or using the automated QIAcube system (Qiagen). RNasin plus RNase inhibitor was added to the RNA (Promega Corporation). Two preparations of 2 μg of RNA from each biopsy were reverse-transcribed in a total volume of 20 μl each using the High-Capacity cDNA Reverse Transcription Kit with RNase inhibitor (Applied Biosystems) according to the manufacturer's instructions. For each biopsy, the resulting cDNA libraries were pooled and stored at −80°C. qPCR was performed using iTaq Universal SYBR Green supermix (Life Technologies) on a ViiA7 Real-Time PCR System (Life Technologies). From the DSS-induced colitis model RNA was purified with lithium chloride as described previously [23], since DSS inhibits reverse transcriptases and polymerases. The ΔΔ*C*_T_ method was used to quantify the mRNA using *TBP* (human) compared with *Tbp* (mouse) as housekeeping genes (*TBP*/*Tbp* encodes TATA-box-binding protein). From Caco-2 cells and macrophages, total RNA was isolated using the Quick-RNA MiniPrep kit (Zymo Research) and reverse-transcribed using the High-Capacity cDNA Reverse Transcription Kit according to the manufacturer's instructions. SYBR Green (Bio-Rad Laboratories) in the 7500-fast-real-time detection system (Applied Biosystems) was used to detect the mRNA levels of *IL34*, *CSF1*, *CSF1R*, *TNFA* and *IL1B* by specific primers (Eurofins) related to the housekeeping genes *ACTB* (encoding β-actin) and *RPL-13a* by the ΔΔ*C*_T_ method. To rule out the possibility of DNA contamination, samples in which the reverse transcription reaction had been omitted were also subjected to PCR, yielding no amplification. Primer sequences are given in Supplementary Table S2.

### Immunohistochemistry

Intestinal biopsies were immediately placed in Histocon solution (Histolab Products) after excision, embedded in optimal cutting temperature compound (OCT; Histolab Products), and kept at −80°C until sectioning. Sections of 7 μm were subjected to fixation in acetone, blocking of endogen peroxidase in methanol and H_2_O_2_ followed by blocking of endogenous alkaline phosphatase by 20% acetic acid. Sections were blocked using avidin and biotin solutions (Vector Laboratories) and incubated in appropriate serum. Anti-IL-34 antibody (catalogue number AB-75723; Abcam) was added, and slides were incubated overnight at 4°C. Sections were incubated with the secondary antibodies using goat anti-rabbit (biotinylated) (catalogue number BA-1000; Vector Laboratories), followed by incubation in Vectastain ABC complex (Vector Laboratories), and development in DAB (diaminobenzidine) solution (Vector Laboratories). Sections were dehydrated and mounted in histograde mounting media (Histolab Products). Rabbit IgG control (catalogue number I-1000; Vector Laboratories) was used as isotype control.

### Immunofluorescence

Immunofluorescent staining was performed on Caco-2 cells cultured on chamber slides (Sarstedt) that were fixed in acetone followed by blocking with 10% (v/v) normal goat serum in PBS. Anti-IL-34 antibody (catalogue number AB-75723; Abcam) was added, and slides were incubated overnight at 4°C. Chamber slides were incubated with secondary goat anti-rabbit IgG conjugated with Alexa Fluor® 488 (catalogue number A11034; Invitrogen), diluted in blocking buffer. Sections stained with the secondary antibody alone were used as negative controls. Sections were mounted in ProLong Gold antifade reagent with DAPI (Invitrogen). Slides were scanned on a LSM710 confocal microscope (Zeiss) in single-photon mode.

### Data handling and statistical analysis

Each patient presented one biopsy from ileum, whereas colon biopsies of each patient were one or more. Therefore we calculated the average of all inflamed biopsies and the average of all the non-inflamed biopsies for each patient in colon. Differences between groups of patients or between ileum and colon were determined using a Mann–Whitney *U* test, and between different sites of colon by ANOVA with an LSD (least significant difference) post-hoc test. The Spearman's correlation coefficients were calculated for correlation assessments. For analyses, SPSS (version 19.0; IBM Corporation) was used. Results are presented as means±S.E.M. The significance levels were set to *P*≤0.05 (*), 0.01 (**) or 0.001 (***).

## RESULTS

### *IL34*, *CSF1* and *CSF1R* are differently expressed in normal human ileum and colon

**Figure 1:**
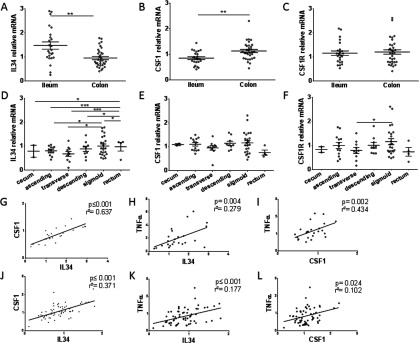
*IL34*, *CSF1* and *CSF1R* are differently expressed in normal human ileum and colon (**A**) *IL34*, (**B**) *CSF1* and (**C**) *CSF1R* relative mRNA expression in ileum and colon, presented as the mean per colon sites for each patient. (**D**) *IL34*, (**E**) *CSF1* and (**F**) *CSF1R* relative mRNA in different sites of the colon. Correlations between (**G** and **J**) *IL34* and *CSF1*, (**H** and **K**) *IL34* and *TNFA*, and (**I** and **L**) *CSF1* and *TNFA* in ileum (**G**–**I**) and colon (**J**–**L**). Comparisons between ileum and colon were calculated using Mann–Whitney *U* tests, and between different sites of colon by ANOVA with post-hoc LSD tests. Correlations were assessed by Spearman's correlation coefficients. *n*=24 for ileum, *n*=29 for colon. Results are means±S.E.M. *P≤0.05; **P≤0.01; ***P≤0.001.

To assess whether the expression of *IL34*, *CSF1* and *CSF1R* differs in human normal ileum and colon, we analysed their mRNA expression in biopsies from 33 control subjects without intestinal inflammation or any other pathological findings. The expression of *IL34* was significantly higher in ileum compared with colon (Figure 1A), whereas, in contrast, higher *CSF1* expression was detected in colon compared with ileum (Figure 1B). *CSF1R* was equivalently expressed in ileum and colon (Figure 1C). Subsequently, we sought further to assess the expression in different regions of the colon. *IL34* expression was significantly higher in rectum compared with sigmoid, descending, transverse, ascending and caecum, and *IL34* expression was higher in sigmoid compared with transverse and ascending colon (Figure 1D). There were no regional differences in the expression of *CSF1* in human normal colon (Figure 1E), whereas *CSF1R* expression was higher in sigmoid compared with transverse colon (Figure 1F). *IL34* and *CSF1* correlated positively with each other and with *TNFA* in ileum (Figures 1G–1I) and colon (Figures 1J–1L). There were no differences in *IL34*, *CSF1* or *CSF1R* expression with respect to gender or age (results not shown).

### Increased expression of *IL34*, *CSF1* and *CSF1R* in inflamed compared with non-inflamed colon of patients with IBD

**Figure 2:**
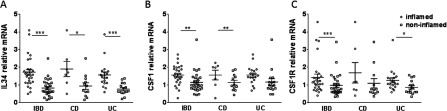
Increased expression of *IL34*, *CSF1* and *CSF1R* in inflamed compared with non-inflamed colon of patients with IBD (**A**) *IL34*, (**B**) *CSF1* and (**C**) *CSF1R* relative mRNA expression in colon presented as the mean per colon sites for each patient in IBD patients subdivided into CD and UC. Comparisons were calculated by Mann–Whitney *U* tests. *n*=21 for non-inflamed IBD, *n*=34 for inflamed IBD, *n*=13 for non-inflamed CD, *n*=8 for non-inflamed CD, *n*=17 for non-inflamed UC, *n*=24 for inflamed UC. Results are means±S.E.M. *P≤0.05; **P≤0.01; ***P≤0.001.

We then investigated the expression of *IL34*, *CSF1* and *CSF1R* in inflamed colon of IBD patients. Strikingly, inflamed colonic areas of IBD patients showed a significant increase in the expression of *IL34*, *CSF1* and *CSF1R* compared with non-inflamed colon (Figures 2A–2C). The difference in *IL34* was evident also when subdividing the IBD patients into CD and UC (Figure 2A), whereas *CSF1* was higher in the inflamed compared with non-inflamed colon of CD patients only (Figure 2B), and *CSF1R* was higher in the inflamed compared with non-inflamed colon of UC patients (Figure 2C). There were no differences for *IL34* or *CSF1* in non-inflamed regions of ileum and colon between IBD patients and controls (Supplementary Figures S1A and S1B and S1D and S1E respectively). The expression of *CSF1R* was significantly lower in CD compared with controls in ileum (Supplementary Figure S1C), and lower in the IBD group and UC compared with controls in colon (Supplementary Figure S1F).

### Presence of IL-34 in the intestinal epithelium and NF-κB-dependent regulation of TNF-α-induced *IL34* and *CSF1* expression in colon epithelial cells

**Figure 3:**
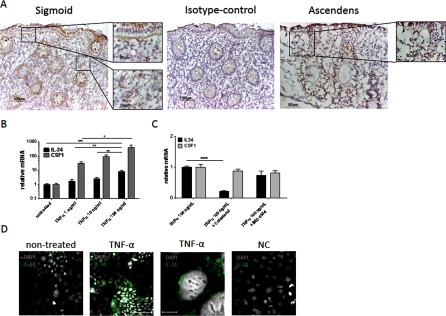
Presence of IL-34 in gut epithelium, and NF-κB-dependent regulation of TNF-α-induced *IL34* and *CSF1* expression in colon epithelial cells (**A**) Immunohistochemical staining of human colon showing the presence of IL-34 and isotype control. (**B**) TNF-α (1–100 ng/ml) up-regulates *IL34* gene expression at 6 h. (**C**) Blocking NF-κB with celastrol down-regulates *IL34* expression in colon epithelial cells. (**D**) The presence of IL-34 shown by immunofluorescent staining of intestinal epithelial cells following TNF-α stimulation. Comparisons were calculated by ordinary one-way ANOVA. Results are means±S.E.M. for two to four individual experiments. *P≤0.05; **P≤0.01; ***P≤0.001.

Given the finding of *IL34* expression in the gut, we next investigated the localization of IL-34 in colon tissue using immunohistochemistry. A positive signal of IL-34 was detected in the epithelial layer; additionally, IL-34-expressing cells were detected in the connective tissue suspected to be infiltrating immune cells (Figure 3A). TNF-α induces the IL-34 expression in fibroblasts and anti-TNF-α inhibits its expression *in vivo* [19,22]. We therefore assessed whether intestinal epithelial cells (Caco-2 cells) express *IL34* and *CSF1* and whether the expression was regulated by TNF-α. For *IL34*, a dose-dependent increase was detected following TNF-α stimulation with the highest expression observed at 100 ng/ml. Also, *CSF1* expression was dose-dependently increased (Figure 3B). The expression of IL-34 was also detected at the protein level by immunofluorescent staining of intestinal epithelial cells; the higher magnification suggests IL-34 expression around the nucleus (Figure 3D). To determine the intracellular signalling involved in TNF-α-induced *IL34* and *CSF1* expression in intestinal epithelial cells, we next used pharmacological inhibitors of NF-κB, a transcription factor downstream of the TNF-α receptor. Treatment with celastrol, an inhibitor of NF-κB, resulted in 80% inhibition of TNF-α-induced *IL34*, and treatment with the inhibitor of the downstream IKKβ [IκB (inhibitor of NF-κB) kinase β] resulted in 25% inhibition of TNF-α-induced *IL34*, which did not reach statistical significance. We found no significant differences in the expression of *CSF1* after blocking NF-κB by the NF-κB inhibitor or the IKKβ inhibitor (Figure 3C).

### IL-34- and CSF-1-differentiated macrophages show decreased *TNFA* and *IL1B* expression, whereas pro-inflammatory cytokines are increased in monocytes from patients with IBD

**Figure 4:**
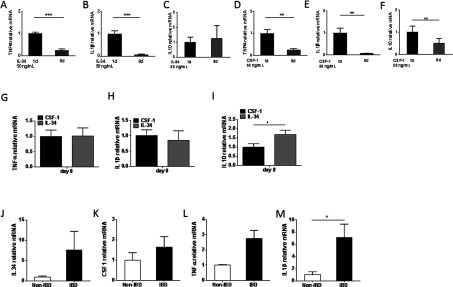
IL-34- and CSF-1-differentiated macrophages show regulated expression of *TNFA, IL1B* and *IL-10*, whereas pro-inflammatory cytokines are increased in monocytes from patients with IBD Regulated expression of *TNFA*, *IL1B* and *IL10* by (**A**–**C**) IL-34 and (**D**–**F**) CSF-1 induced differentiation of macrophages (day 8). Expression of (**G**) *TNFA*, (**H**) *IL1B* and (**I**) *IL10* in CSF-1- and IL-34-differentiated macrophages. Expression of (**J**) *IL34*, (**K**) *CSF1*, (**L**) TNFA and (**M**) IL1B in monocytes from IBD patients and controls. The differentiation experiment was performed twice with similar results. *n*=6 for control monocytes, *n*=10 for IBD-monocytes. Results are means±S.E.M. *P≤0.05; **P≤0.01; ***P≤0.001.

Given the finding that intestinal epithelial cells express *IL34* and *CSF1* and that IL-34 and CSF-1 are key regulators of monocytes and macrophages, we next investigated the expression of pro-inflammatory cytokines in monocytes differentiated with IL-34 or CSF-1. Following differentiation at day 8 (fully differentiated macrophages), the expression of both *TNFA* and *IL1B* was significantly decreased by IL-34, and *TNFA*, *IL1B* and *IL10* were significantly decreased by CSF-1 (Figures 4A–4F). There was no difference in the expression of *TNFA* or *IL1B* between IL-34- and CSF-1-differentiated macrophages, but *IL10* was significantly higher in the IL-34-differentiated cells (Figures 4G–4I). Since monocytes frequently enter the intestine in IBD, we next compared the expression of pro-inflammatory cytokines in monocytes from patients with IBD and from healthy donors as controls. Compared with healthy donors, monocytes from IBD patients showed an increased expression of *IL1B*, and a tendency for increased expression of *TNFA* and *CSF1* which, however, did not reach statistical significance (*P*=0.19 compared with *P*=0.37). We did not detect any difference in the expression of *IL34* in monocytes (Figures 4E–4H).

### Increased expression of *Il34* and *Csf1* in DSS-induced colitis

**Figure 5:**
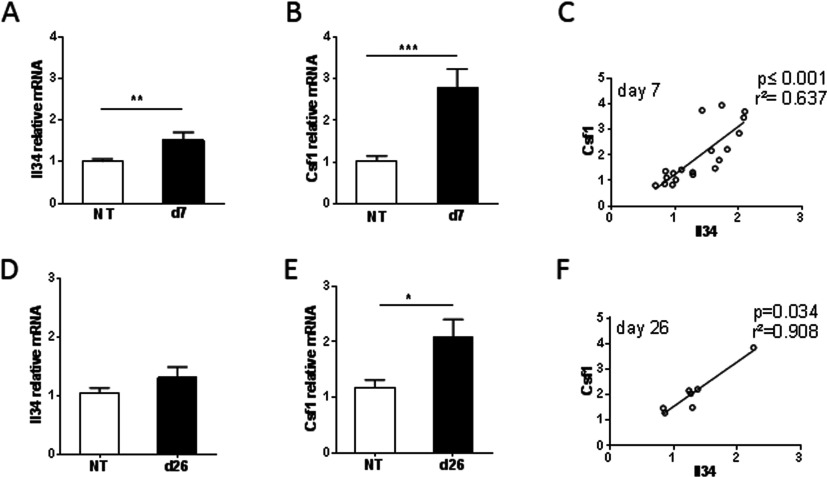
Increased expression of *Il34* and *Csf1* in DSS-induced colitis in mice (**A**) *Il34* and (**B**) *Csf1* relative mRNA expression in colon on day 5. (**C**) Correlation between *Il34* and *Csf1* in colons of DSS-treated mice on day 5. (**D**) *Il34* (**E**) *Csf1* relative mRNA expression in colon on day 26. (**F**) Correlation between *Il34* and *Csf1* in colons of DSS-treated mice on day 26. The experiment was performed twice with similar results. Results are means±S.E.M. Correlations were assessed by Spearman's correlation coefficients. NT, non-treated. *P≤0.05; **P≤0.01; ***P≤0.001.

Mice lacking one allele of the CSF-1R are protected from chemically induced colitis, indicating a potential beneficial effect of CSF-1R ligands in colonic inflammation [24]. In the light of the increased *IL34* and *CSF1* expression in the inflamed intestine of patients with IBD, we next assessed their involvement in the widely used DSS model of murine colitis [25]. At day 7 after DSS administration, in a state of acute epithelial damage and severe acute intestinal inflammation, both *Il34* and *Csf1* were significantly up-regulated in the colon (Figures 5A and 5B respectively), and correlated positively with each other (Figure 5C). We evaluated, further, their expression at a later time point (21 days after DSS was stopped), in which inflammatory infiltrates remain paralleled to enhanced epithelial regeneration processes [26]. At this later time point, *Csf1* was continuously up-regulated; however, just a tendency of *Il34* up-regulation was measured, which did not reach statistical significance.

## DISCUSSION

Macrophages are functionally dependent on CSF-1 or the newly identified factor IL-34 and the intestines represent the largest reservoir of macrophages of the body [14]. Despite an important role of macrophages in the course of IBD, the expression and relevance of intestinal IL-34 in health and disease is until now unknown. In the present study, we demonstrate distinct expression patterns of *IL34* and *CSF1* in human normal intestine, regulation of *IL34* and *CSF1* with inflammation in human IBD and a mouse model of colitis, and identify intestinal epithelial cells as a cellular source of IL-34. This is, to our knowledge, the first study providing evidence for the expression pattern of IL-34 in ileum and colon of healthy subjects and IBD patients.

Compartmental differences between ileum and colon have previously been studied where left colonic segments of CD patients have lesions to a higher extent compared with other segments in colon [27]. In human normal intestine, we found higher *IL34* in ileum and, in contrast, higher *CSF1* in colon. Moreover, *IL34* expression was highest in the left colonic segments with highest expression in the rectum, followed by the sigmoid colon. This is also interesting in relation to reported regional differences in macrophages between segments [28].

We found an increase in the expression of *IL34* and *CSF1* with inflammation in CD and UC patients. This is intriguing as IL-34 has been associated with local inflammation in other chronic inflammatory diseases including RA and Sjögren's syndrome [19,20]. It is known that the composition of intestinal macrophages changes with inflammation and CD14^high^ macrophages that produce TNF-α, IL-1, IL-6 and nitric oxide accumulate [9,29]. As these cells may be of importance for therapeutic targeting, it is important to establish whether they represent monocyte-derived cells and not altered resident macrophages. The pro- or anti-inflammatory role of IL-34 is until now unknown. We found that intestinal epithelial cells produce *IL34* and *CSF1*, and that monocytes differentiated by IL-34 and CSF-1 decrease their expression of *TNFA* and *IL1B*. Moreover, IL-34-differentiated macrophages expressed significantly more IL-10 compared with macrophages differentiated by CSF-1. This is interesting as it suggests a potential role for IL-34 in stimulating the differentiation towards resident macrophages, which contribute to the integrity of the intestinal epithelium, and enhanced expression of IL-34 could reflect a need to increase the pool of resident macrophages in order to maintain the intestinal barrier function. Further studies are, however, needed to investigate whether these cells would originate from circulating monocytes, especially in the light of previously published work showing that many membrane-bound receptors are absent from intestinal macrophages compared with blood monocytes [8].

TNF-α is one of the most potent pro-inflammatory mediators in IBD pathogenesis. Anti-TNF-α treatment reduces inflammation and can induce and maintain remission in patients with CD and UC [30–36]. TNF-α has also been shown to disrupt the intestinal epithelial barrier by degradation of the tight junctions [11,37]. In the present study, we show expression of *IL34* in the intestinal epithelium and demonstrate increased *IL34* and *CSF1* expression in intestinal epithelial cells in response to TNF-α. To date, no previous studies have identified IL-34 expression and regulation in gut epithelial cells. Skin keratinocytes represent one of the major cellular sources of IL-34 in mice [18] and expression in human skin keratinocytes was shown recently [38]. Interestingly, IL-34 expression in skin was decreased in lesional skin of AD (atopic dermatitis) patients [38], described to be related to the skin barrier, which is affected in AD. This is, despite the large differences in the epithelium of the skin and intestine, interesting as impaired epithelial barrier is also a hallmark of inflamed tissues in IBD patients where we find an increase in *IL34* expression. This could possibly partly be explained by differences in the cytokine environment where AD is a Th2/Th22-induced inflammatory skin disease, whereas RA and IBD are more closely related to a more complex Th1/Th17 cytokine milieu (reviewed in [39,40]). Nevertheless, identification of IL-34 expression in skin keratinocytes supports our finding that IL-34 is expressed by resident non-immune cells of tissues, supported further by previous studies by our group and others that identify IL-34 expression in fibroblasts [21,38].

Blocking the NF-κB pathway resulted in reduced TNF-α-stimulated *IL34* expression in colon epithelial cells. Since macrophages in IBD express pro-inflammatory cytokines, TNF-α from macrophages could induce IL-34 and CSF-1 expression in epithelial cells. Several studies have investigated the interaction between macrophages and epithelial cells in IBD. Depletion of macrophages and dendritic cells worsened the histopathology with crypt destruction and epithelial disruption in experimental colitis [41]. Furthermore, macrophages promote proliferation and survival of colonic epithelial progenitor cells independently of lymphocytes or neutrophils [42]. Additionally, defective proliferation of colon epithelial cells is described in the *Csf1r*^−/−^ and *Csf1*^op/op^ mice and reduced expression of cell cycle genes is measured in crypts of the *Csf1r*-deficient mice [24]. Thus, CSF-1 and also IL-34 may have protective functions by recruiting tissue macrophages, which, compared with monocyte-derived pro-inflammatory macrophages, do not respond to inflammation but promote cell proliferation and survival of colon epithelial cells [8,9,24].

In conclusion, we show for the first time the expression pattern of IL-34 in human normal intestine and in human and experimental IBD. We identify intestinal epithelial cells regulated by TNF-α as a cellular source of IL-34. The present study provides evidence for the expression and a potential role for IL-34 in IBD.

## CLINICAL PERSPECTIVES

• Inflammatory bowel disease (IBD) is a chronic inflammatory condition of the intestines where macrophages play a central role. Macrophages depend on CSF-1 and IL-34; however, until now, the expression and regulation of IL-34 in the gut is unknown.
• We show distinct expression patterns of *IL34* and *CSF1* in human normal intestine, expression in intestinal epithelium, and increased expression in human and experimental IBD.
• This provides evidence for the involvement of IL-34 in IBD and may lead to new therapeutic strategies modulating intestinal macrophages.

## Online data

Supplementary data

## References

[1] Krishnan K., Arnone B., Buchman A. (2011). Intestinal growth factors: potential use in the treatment of inflammatory bowel disease and their role in mucosal healing. Inflamm. Bowel Dis..

[2] Ordas I., Eckmann L., Talamini M., Baumgart D.C., Sandborn W.J. (2012). Ulcerative colitis. Lancet.

[3] Baumgart D.C., Sandborn W.J. (2012). Crohn's disease. Lancet.

[4] Chow A., Brown B.D., Merad M. (2011). Studying the mononuclear phagocyte system in the molecular age. Nat. Rev. Immunol..

[5] van Furth R., Cohn Z.A., Hirsch J.G., Humphrey J.H., Spector W.G., Langevoort H.L. (1972). The mononuclear phagocyte system: a new classification of macrophages, monocytes, and their precursor cells. Bull. World Health Organ..

[6] Hettinger J., Richards D.M., Hansson J., Barra M.M., Joschko A.C., Krijgsveld J., Feuerer M. (2013). Origin of monocytes and macrophages in a committed progenitor. Nat. Immunol..

[7] Anderson K.L., Smith K.A., Conners K., McKercher S.R., Maki R.A., Torbett B.E. (1998). Myeloid development is selectively disrupted in PU.1 null mice. Blood.

[8] Smythies L.E., Sellers M., Clements R.H., Mosteller-Barnum M., Meng G., Benjamin W.H., Orenstein J.M., Smith P.D. (2005). Human intestinal macrophages display profound inflammatory anergy despite avid phagocytic and bacteriocidal activity. J. Clin. Invest..

[9] Bain C.C., Scott C.L., Uronen-Hansson H., Gudjonsson S., Jansson O., Grip O., Guilliams M., Malissen B., Agace W.W., Mowat A.M. (2013). Resident and pro-inflammatory macrophages in the colon represent alternative context-dependent fates of the same Ly6Chi monocyte precursors. Mucosal Immunol..

[10] Rugtveit J., Nilsen E.M., Bakka A., Carlsen H., Brandtzaeg P., Scott H. (1997). Cytokine profiles differ in newly recruited and resident subsets of mucosal macrophages from inflammatory bowel disease. Gastroenterology.

[11] Vivinus-Nebot M., Frin-Mathy G., Bzioueche H., Dainese R., Bernard G., Anty R., Filippi J., Saint-Paul M.C., Tulic M.K., Verhasselt V. (2014). Functional bowel symptoms in quiescent inflammatory bowel diseases: role of epithelial barrier disruption and low-grade inflammation. Gut.

[12] Van Deventer S.J. (1997). Tumour necrosis factor and Crohn's disease. Gut.

[13] Dai X.M., Ryan G.R., Hapel A.J., Dominguez M.G., Russell R.G., Kapp S., Sylvestre V., Stanley E.R. (2002). Targeted disruption of the mouse colony-stimulating factor 1 receptor gene results in osteopetrosis, mononuclear phagocyte deficiency, increased primitive progenitor cell frequencies, and reproductive defects. Blood.

[14] Lin H., Lee E., Hestir K., Leo C., Huang M., Bosch E., Halenbeck R., Wu G., Zhou A., Behrens D. (2008). Discovery of a cytokine and its receptor by functional screening of the extracellular proteome. Science.

[15] Wei S., Nandi S., Chitu V., Yeung Y.G., Yu W., Huang M., Williams L.T., Lin H., Stanley E.R. (2010). Functional overlap but differential expression of CSF-1 and IL-34 in their CSF-1 receptor-mediated regulation of myeloid cells. J. Leukoc. Biol..

[16] Ma X., Lin W.Y., Chen Y., Stawicki S., Mukhyala K., Wu Y., Martin F., Bazan J.F., Starovasnik M.A. (2012). Structural basis for the dual recognition of helical cytokines IL-34 and CSF-1 by CSF-1R. Structure.

[17] Nandi S., Gokhan S., Dai X.M., Wei S., Enikolopov G., Lin H., Mehler M.F., Stanley E.R. (2012). The CSF-1 receptor ligands IL-34 and CSF-1 exhibit distinct developmental brain expression patterns and regulate neural progenitor cell maintenance and maturation. Dev. Biol..

[18] Wang Y., Szretter K.J., Vermi W., Gilfillan S., Rossini C., Cella M., Barrow A.D., Diamond M.S., Colonna M. (2012). IL-34 is a tissue-restricted ligand of CSF1R required for the development of Langerhans cells and microglia. Nat. Immunol..

[19] Chemel M., Le Goff B., Brion R., Cozic C., Berreur M., Amiaud J., Bougras G., Touchais S., Blanchard F., Heymann M.F. (2012). Interleukin 34 expression is associated with synovitis severity in rheumatoid arthritis patients. Ann. Rheum. Dis..

[20] Ciccia F., Alessandro R., Rodolico V., Guggino G., Raimondo S., Guarnotta C., Giardina A., Sireci G., Campisi G., De Leo G. (2013). IL-34 is overexpressed in the inflamed salivary glands of patients with Sjögren's syndrome and is associated with the local expansion of pro-inflammatory CD14^bright^CD16^+^ monocytes. Rheumatology.

[21] Bostrom E.A., Lundberg P. (2013). The newly discovered cytokine IL-34 is expressed in gingival fibroblasts, shows enhanced expression by pro-inflammatory cytokines, and stimulates osteoclast differentiation. PLoS ONE.

[22] Tian Y., Shen H., Xia L., Lu J. (2013). Elevated serum and synovial fluid levels of interleukin-34 in rheumatoid arthritis: possible association with disease progression via interleukin-17 production. J. Interferon Cytokine Res..

[23] Viennois E., Chen F., Laroui H., Baker M.T., Merlin D. (2013). Dextran sodium sulfate inhibits the activities of both polymerase and reverse transcriptase: lithium chloride purification, a rapid and efficient technique to purify RNA. BMC Res. Notes.

[24] Huynh D., Akcora D., Malaterre J., Chan C.K., Dai X.M., Bertoncello I., Stanley E.R., Ramsay R.G. (2013). CSF-1 receptor-dependent colon development, homeostasis and inflammatory stress response. PLoS ONE.

[25] Wirtz S., Neufert C., Weigmann B., Neurath M.F. (2007). Chemically induced mouse models of intestinal inflammation. Nat. Protoc..

[26] Melgar S., Karlsson A., Michaelsson E. (2005). Acute colitis induced by dextran sulfate sodium progresses to chronicity in C57BL/6 but not in BALB/c mice: correlation between symptoms and inflammation. Am. J. Physiol. Gastrointest. Liver Physiol..

[27] Maccioni F., Viola F., Carrozzo F., Di Nardo G., Pino A.R., Staltari I., Al Ansari N., Vestri A., Signore A., Marini M. (2012). Differences in the location and activity of intestinal Crohn's disease lesions between adult and paediatric patients detected with MRI. Eur. Radiol..

[28] Denning T.L., Norris B.A., Medina-Contreras O., Manicassamy S., Geem D., Madan R., Karp C.L., Pulendran B. (2011). Functional specializations of intestinal dendritic cell and macrophage subsets that control Th17 and regulatory T cell responses are dependent on the T cell/APC ratio, source of mouse strain, and regional localization. J. Immunol..

[29] MacDonald T.T., Monteleone I., Fantini M.C., Monteleone G. (2011). Regulation of homeostasis and inflammation in the intestine. Gastroenterology.

[30] Kriegel C., Amiji M. (2011). Oral TNF-α gene silencing using a polymeric microsphere-based delivery system for the treatment of inflammatory bowel disease. J. Control. Release.

[31] Hanauer S.B., Feagan B.G., Lichtenstein G.R., Mayer L.F., Schreiber S., Colombel J.F., Rachmilewitz D., Wolf D.C., Olson A., Bao W. (2002). Maintenance infliximab for Crohn's disease: the ACCENT I randomised trial. Lancet.

[32] Cohen R.D., Tsang J.F., Hanauer S.B. (2000). Infliximab in Crohn's disease: first anniversary clinical experience. Am. J. Gastroenterol..

[33] Armuzzi A., De Pascalis B., Fedeli P., De Vincentis F., Gasbarrini A. (2008). Infliximab in Crohn's disease: early and long-term treatment. Dig. Liver Dis..

[34] D’Haens G., Baert F., van Assche G., Caenepeel P., Vergauwe P., Tuynman H., De Vos M., van Deventer S., Stitt L., Donner A. (2008). Early combined immunosuppression or conventional management in patients with newly diagnosed Crohn's disease: an open randomised trial. Lancet.

[35] Lichtenstein G.R., Diamond R.H., Wagner C.L., Fasanmade A.A., Olson A.D., Marano C.W., Johanns J., Lang Y., Sandborn W.J. (2009). Clinical trial: benefits and risks of immunomodulators and maintenance infliximab for IBD-subgroup analyses across four randomized trials. Aliment. Pharmacol. Ther..

[36] Panaccione R., Ghosh S., Middleton S., Marquez J.R., Scott B.B., Flint L., van Hoogstraten H.J., Chen A.C., Zheng H., Danese S. (2014). Combination therapy with infliximab and azathioprine is superior to monotherapy with either agent in ulcerative colitis. Gastroenterology.

[37] He F., Peng J., Deng X.L., Yang L.F., Camara A.D., Omran A., Wang G.L., Wu L.W., Zhang C.L., Yin F. (2012). Mechanisms of tumor necrosis factor-alpha-induced leaks in intestine epithelial barrier. Cytokine.

[38] Esaki H., Ewald D.A., Ungar B., Rozenblit M., Zheng X., Xu H., Estrada Y.D., Peng X., Mitsui H., Litman T. (2015). Identification of novel immune and barrier genes in atopic dermatitis by means of laser capture microdissection. J. Allergy Clin. Immunol..

[39] Wallace K.L., Zheng L.B., Kanazawa Y., Shih D.Q. (2014). Immunopathology of inflammatory bowel disease. World J. Gastroenterol..

[40] Cosmi L., Liotta F., Maggi E., Romagnani S., Annunziato F. (2014). Th17 and non-classic Th1 cells in chronic inflammatory disorders: two sides of the same coin. Int. Arch. Allergy Immunol..

[41] Qualls J.E., Kaplan A.M., van Rooijen N., Cohen D.A. (2006). Suppression of experimental colitis by intestinal mononuclear phagocytes. J. Leukoc. Biol..

[42] Pull S.L., Doherty J.M., Mills J.C., Gordon J.I., Stappenbeck T.S. (2005). Activated macrophages are an adaptive element of the colonic epithelial progenitor niche necessary for regenerative responses to injury. Proc. Natl. Acad. Sci. U.S.A..

