# Supplementary material for: Interleukin 34: a new modulator of human and experimental inflammatory bowel disease
Source: Clin Sci (Lond). 2015 May 15;129(Pt 3):281–90. doi: 10.1042/CS20150176 (PMC4557398; doi:10.1042/CS20150176)
Supplement: Supplementary data [file cs1290281ntsadd.pdf]

Supplemental Figure 1.

Expression of IL34, CSF1, and CSF1R in normal human ileum and colon and in non-inflamed ileum and colon of patients with inflammatory bowel disease. (A-F) (A) IL34, (B) CSF1, and (C) CSF1R relative mRNA expression in colon presented as the mean per colon sites for each patient, and in (D) (E) (F) ileum. Differences between groups were assessed by Mann-Whitney U Tests. Ileum: n=24 for non-IBD, n=24 for IBD, n=11 for CD, n=12 for UC. Colon: n=33 for non-IBD, n=29 for IBD, n=12 for CD, n=16 for UC. Data is presented as mean ± SEM. *p≤0.05; **p≤0.01; ***p≤0.001.

Suppl. Fig. 1

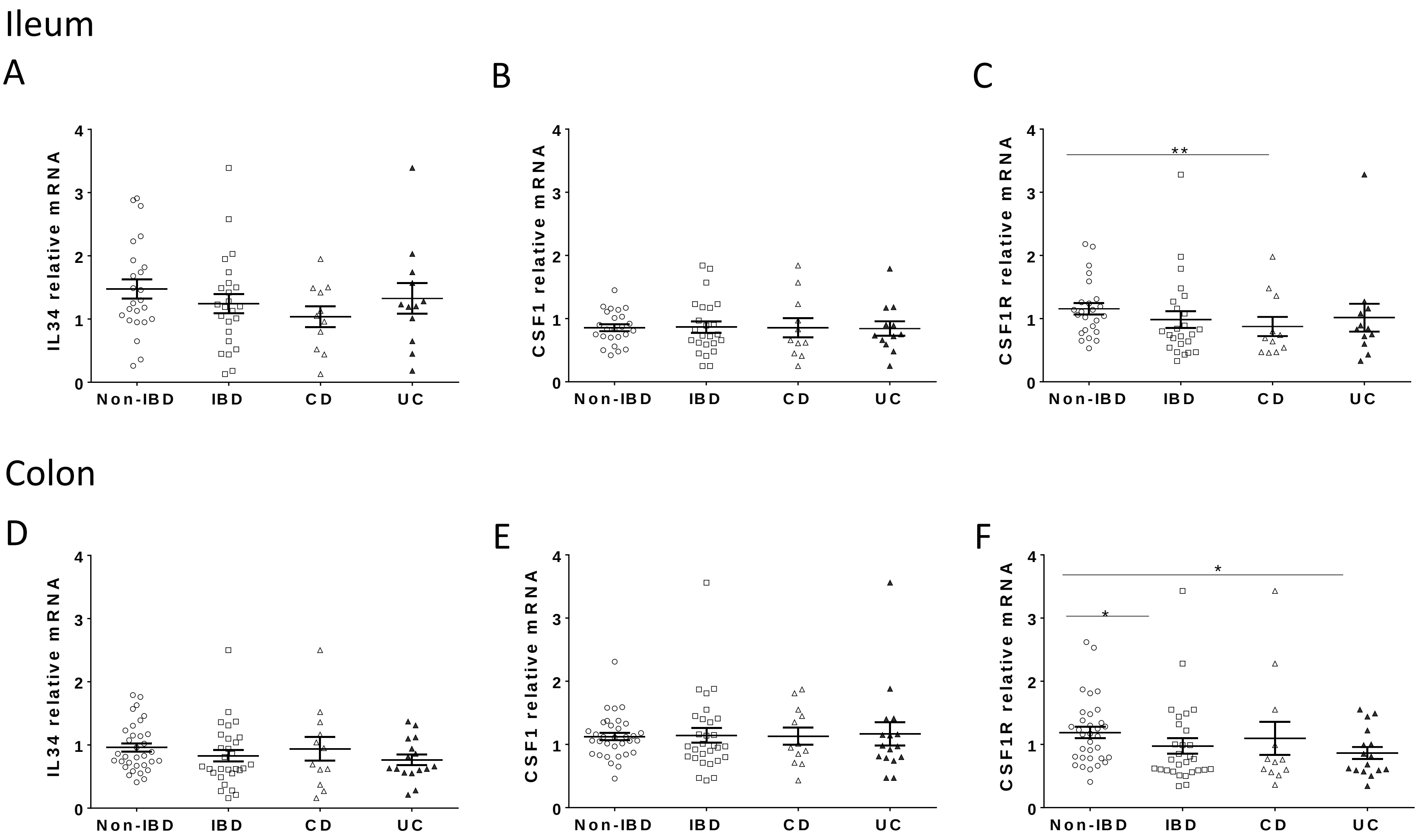

Zwicker et al.

Supplemental Table 1. Characteristics of study participants

	IBD (n=52)	Non-IBD (n=33)
**Disease** (CD/ UC/IBDU)	21/29/2	0
**Gender** (Male/ Female)	23/29	13/20
**Age** (years)[1]	40 (18-77)	49 (20-82)
**Immune-modulating drugs**		
Aminosalicylates	24	0
Thiopurines	17	0
Corticosteroids	17	0
Anti-TNF-α	6	1
Methotrexate	2	0
Tacrolimus	2	0
None	10	32

[1]Median (min-max)

Supplemental table 2. Primer sequences.

	Forward	Reverse
hIL34	GCCACCCATCCTGGAAGTA	GACAACACGGATTCCACCTT
hCSF1	GTGGAACTGCCAGTGTAGAGG	TGGAGGGCAGACCACATT
hCSF1R	ATGCTACCACCAAGGACACA	AGCCTCCTGGGTTTCTGG
hTNFA	GACAAGCCTGTAGCCCATGT	TCTCAGCTCCACGCCATT
hIL1B	TACCTGTCCTGCGTGTTGAA	TCTTTGGGTAATTTTTGGGATCT
hTBP	CCACTCACAGACTCTCACAAC	CTGCGGTACAATCCCAGAACT
hGAPDH	TCCCACTGGCGTCTTCACC	GGCAGAGATGATGACCCTTTT
hRPL13A	CAAGCGGATGAACACCAAC	TGTGGGGCATACCTC
mIl34	TTGCTGTAAACAAAGCCCCAT	CCGAGACAAAGGGTACACATTT
mCsf1	GGCTTGGCTTGGGATGATTCT	GAGGGTCTGGCAGGTACTC
mTbp	GCTCTGGAATTGTACCGCAG	CTGGCTCATAGCTCTTGGCTC
